# RETRACTION: Wound Cosmesis Problems and Other Postoperative Problems of Laparoscopic Compared to Open Paediatric Inguinal Hernia Repair: A Meta‐Analysis

**DOI:** 10.1111/iwj.70206

**Published:** 2025-02-02

## Abstract

**RETRACTION:** LiQ. , LiuS. , MaX. , and YuJ. , “Wound Cosmesis Problems and Other Postoperative Problems of Laparoscopic Compared to Open Paediatric Inguinal Hernia Repair: A Meta‐Analysis,” International Wound Journal 20, no. 9 (2023): 3665–3672, 10.1111/iwj.14257.37303125 PMC10588365

The above article, published online on 11 June 2023, in Wiley Online Library (http://onlinelibrary.wiley.com/), has been retracted by agreement between the journal Editor in Chief, Professor Keith Harding; and John Wiley & Sons Ltd. Following an investigation by the publisher, all parties have concluded that this article was accepted solely on the basis of a compromised peer review process. The editors have therefore decided to retract the article. The authors did not respond to our notice regarding the retraction.

**RETRACTION:** Q. Li , S. Liu , X. Ma , and J. Yu , “Wound Cosmesis Problems and Other Postoperative Problems of Laparoscopic Compared to Open Paediatric Inguinal Hernia Repair: A Meta‐Analysis,” International Wound Journal 20, no. 9 (2023): 3665–3672, 10.1111/iwj.14257.37303125 PMC10588365

The above article, published online on 11 June 2023, in Wiley Online Library (http://onlinelibrary.wiley.com/), has been retracted by agreement between the journal Editor in Chief, Professor Keith Harding; and John Wiley & Sons Ltd. Following an investigation by the publisher, all parties have concluded that this article was accepted solely on the basis of a compromised peer review process. The editors have therefore decided to retract the article. The authors did not respond to our notice regarding the retraction.

